# A PILOT STUDY OF AN INTERGENERATIONAL BOOK CLUB: LESSONS LEARNED FOR IMPROVING FEASIBILITY

**DOI:** 10.1093/geroni/igac059.2760

**Published:** 2022-12-20

**Authors:** Katelyn Frey, Jennifer Sublett, Jennifer Stanley, Harvey Stearns

**Affiliations:** The University of Akron, Akron, Ohio, United States; The University of Akron, Akron, Ohio, United States; The University of Akron, Akron, Ohio, United States; The University of Akron, Akron, Ohio, United States

## Abstract

Intergenerational book clubs have been successful in reducing ageist attitudes of younger adults (YA) while increasing feelings of social connectedness among older adults (OA; Lohman et al., 2003). We implemented both in-person (Nf11: YA=4, OA=7) and virtual (Nf8: YA=5, OA=3) modalities for an intergenerational book club to increase intergenerational interactions on campus, considering aging researchers’ claim of heightened ageism and exacerbated loneliness following the beginning of the COVID-19 pandemic (Ayalon et al., 2020; Brooke & Jackson, 2020). Both groups met thrice over twelve weeks and completed measures on ageist attitudes (α=.83), engagement with the group (α=.91), and social connectedness (α=.91) each time. There was attrition in both groups (final meeting: Nf9 (in-person) and Nf3 (virtual)). To better understand overall motivations to participate and what factors may have contributed to attrition, we utilized content analysis with participants’ feedback to examine their motivation for joining, what they enjoyed most after participating, and what they would improve. Participants were motivated to join because they enjoyed reading; comparatively, they enjoyed group discussions and hearing different perspectives. Future recommendations include meeting more often, increasing the number and size of groups, and scheduling meetings closer together. The majority wished to continue participating. The data suggest future expansion of intergenerational book clubs on campus are desired to support age-friendly interactions and needed to further examine if these interactions can curb ageism and increase social connectedness among diverse age groups. Discussion will include recommendations regarding measures, lessons learned for an optimal protocol, and next steps.

